# Setting of Cement

**Published:** 1904-11

**Authors:** 


Setting of Cement.—In connection with the powder portion, there are two conditions which markedly influence the setting—viz., fineness of division and pigmenting. A finely divided powder gives more rapid chemical action, simply because of exposing more surface to the reagent, and pigmenting hastens or retards setting according as the pigment tends to form basic phosphate more or less rapidly It can be said that most oxides used for pigmenting have the tendency to retard setting, so that yellow, brown and gray powders can be depended on for slower setting than white.

To sum up, the setting of a oxphosphate can be hastened by the addition of water or diluted phosphoric acid to the liquid, or by reducing the powder to a more finely divided state. The setting can be retarded by abstracting water from the liquid, or by adding phosphate ("flux") to the liquid, or by pigmenting of the powder with oxides tending to form basic phosphate slowly.—*West. Jour.*

